# The Effects of Different Companion Fungi on the Growth and Development of *Cremastra appendiculata* and the Accumulation of Bioactive Components in Its Pseudobulbs

**DOI:** 10.3390/plants15193012

**Published:** 2026-09-30

**Authors:** Siwen Wang, Liang Wang, Jingjing Li, Yuhe Xing, Jie Wang, Jinghan Wang, Xinyue Hou, Yue Zhang, Bingyan Liu

**Affiliations:** 1 College of Landscape Architecture, Changchun University, Changchun 130022, China; 13630643305@163.com (S.W.); 18330815511@163.com (J.L.); 17504456087@163.com (Y.X.); 15829539117@163.com (J.W.); 19508689752@163.com (X.H.); 15585665610@163.com (Y.Z.); 2 Jilin Academy of Agricultural Sciences (Northeast Agricultural Research Center of China), Changchun 130033, China; wangliang8008@126.com; 3 College of Health Technology Industry, Jilin Science and Technology Vocational College, Changchun 130123, China; 15144131788@163.com

**Keywords:** *Cremastra appendiculata*, companion fungi, pseudobulb, growth and development, bioactive components, comprehensive evaluation

## Abstract

*Cremastra appendiculata* (D. Don) Makino is a rare orchid species possessing both ornamental and medicinal value. The process by which its protocorms develop into pseudobulbs represents not only a critical stage for seedling establishment but also a key period for the formation and accumulation of pharmacologically active constituents. Mycorrhizal fungi and plant growth-promoting microorganisms are known to promote the growth and development of orchids; however, their integrated regulatory effects on pseudobulb growth and quality formation in *C. appendiculata* remain unclear. In this study, pseudobulbs of *C. appendiculata* were used as experimental materials and treated with three companion fungal inoculants, namely *Coprinellus micaceus*, *Coprinellus radians*, and *Ephemerocybe pseudodisseminata*. Since *C. appendiculata* seeds cannot germinate under sterile conditions, no sterile control group was included in this study; therefore, the results primarily reflect differences among the three fungal treatments rather than absolute growth-promoting effects. Subsequently, plant growth parameters, biomass indices, physiological and biochemical traits, and contents of medicinal quality components were measured, and the comprehensive effects of different companion fungi were evaluated using principal component analysis (PCA) and correlation analysis. The results showed that plants treated with *C. micaceus* had significantly higher plant height, stem base diameter, leaf area, main root length, and pseudobulb length and width, as well as higher fresh and dry weights of pseudobulbs, compared with the other two treatments. In terms of physiological and biochemical indices, the contents of soluble protein, starch, and crude ash in pseudobulbs were highest under *C. micaceus* treatment. Quality component analysis revealed that the contents of total flavonoids, total phenolics, polysaccharides, and alkaloids were significantly higher under *C. micaceus* treatment than under the other treatments, with the alkaloid content reaching 1.66 mg BE·g^−1^. Principal component analysis indicated that the cumulative contribution rate of PC1 and PC2 reached 95.4%, effectively explaining the variation among different treatments. The comprehensive evaluation results demonstrated that the effects of the different companion fungal treatments ranked as follows: *C. micaceus* > *C. radians* > *E. pseudodisseminata*. In conclusion, among the three companion fungal treatments, *C. micaceus* showed the best comprehensive performance in promoting pseudobulb growth and medicinal quality formation in *C. appendiculata*. This fungus can serve as an excellent microbial inoculant for seedling propagation and efficient cultivation of medicinal resources of *C. appendiculata*, thereby providing a theoretical basis for the artificial cultivation and resource exploitation of this species.

## 1. Introduction

*Cremastra appendiculata* (D. Don) Makino, a rare perennial medicinal plant belonging to the genus *Cremastra* Lindl. within the family Orchidaceae, possesses significant medicinal and ornamental value [1] and is widely distributed in East Asian countries including China, Japan, and the Republic of Korea. Its dried pseudobulbs serve as one of the important sources of the traditional Chinese medicine *Shancigu*, which exhibits pharmacological effects such as heat-clearing and detoxification, swelling reduction, and antitumor activity, conferring substantial application value in clinical practice and natural drug development [2]. In recent years, owing to increasing market demand and excessive wild resource harvesting, the natural population size of *C. appendiculata* has been continuously declining, rendering it an important medicinal plant in urgent need of conservation and efficient propagation [3].

Nevertheless, large-scale artificial cultivation of *C. appendiculata* faces multiple biological bottlenecks. Studies have revealed that the seeds of *C. appendiculata* are minute and lack endosperm, resulting in low germination rates under natural conditions [4]. Additionally, the renewal rate of pseudobulbs is slow, with typically only one new pseudobulb formed per year [5]. More critically, pseudobulbs, as the primary medicinal organ of *C. appendiculata*, are rich in various bioactive constituents, including polysaccharides, flavonoids, phenolics, alkaloids, bibenzyls, and phenanthrenes [6]. The synergistic enhancement of both growth rate and medicinal quality poses a core challenge for the industrialized exploitation of these resources. Therefore, how to promote pseudobulb growth while simultaneously enhancing the accumulation of medicinal active ingredients has become an important research direction in the conservation and utilization of *C. appendiculata* resources.

Mycorrhizal fungi and plant growth-promoting microorganisms can significantly facilitate the growth and development of orchids through various mechanisms, including promoting mineral nutrient uptake, regulating plant hormone balance, enhancing carbon and nitrogen metabolism [7], and improving stress adaptability [8]. In recent years, microbial regulation has emerged as an important technical approach for quality enhancement in medicinal plants. For *C. appendiculata*, existing research has primarily focused on the promoting effects of mycorrhizal fungi on seed germination and seedling establishment. Gao et al. [9] first reported that *Coprinellus disseminatus* could promote seed germination of *C. appendiculata* (achieving a germination rate of 71.61% within 30 days), with seedlings possessing one leaf and one root forming after approximately six months. Xiao et al. [1] further demonstrated that *Coprinellus radians* could increase the seed germination rate to over 75% on OMA medium, and morphological and histological observations revealed that hyphae first penetrated the embryo cells and subsequently colonized the rhizome cells of the seedlings. Shi et al. [10] found that the widely distributed *Coprinellus micaceus* promoted seed germination of *C. appendiculata* in both culture medium and soil, with protocorms capable of digesting the invading hyphae. At the molecular level, Gao et al. [9] revealed through biochemical and transcriptomic analyses that *C. disseminatus* promotes water absorption by degrading lignocellulose in the seed coat (with lignin content decreasing by 73% and water content increasing from 13% to 90%) and regulates the expression of genes related to abscisic acid biosynthesis, thereby breaking seed dormancy. Collectively, these studies indicate that specific fungi play an indispensable growth-promoting role in seed germination of *C. appendiculata*.

Notably, as the core organ governing the medicinal quality formation of *C. appendiculata*, the accumulation of bioactive components in pseudobulbs depends not only on biomass increase but also, more critically, on the activation and regulation of secondary metabolic pathways [11]. Previous studies have demonstrated that mycorrhizal fungi and plant growth-promoting microorganisms can act as biological elicitors to activate secondary metabolic pathways and promote the accumulation of bioactive substances such as flavonoids, phenolics, polysaccharides, and alkaloids [12]. For medicinal orchids, microbial regulation has become an important technical approach for enhancing medicinal material quality [13]. However, current research on companion fungal inoculants for *C. appendiculata* has almost entirely focused on seed germination rate and seedling establishment rate, whereas systematic studies regarding their integrated regulatory effects on pseudobulb growth and quality formation are still lacking. In particular, there is a notable absence of comprehensive evaluations of different companion fungi from multiple perspectives, including growth and development, physiological and biochemical characteristics, and bioactive component accumulation. Given that pseudobulbs serve as the primary medicinal part of *C. appendiculata*, their quality directly determines the clinical value and economic benefits of the medicinal material. Therefore, screening for superior companion fungi that can both promote pseudobulb growth and enhance bioactive component accumulation holds significant theoretical and practical importance for the artificial cultivation and efficient medicinal resource development of *C. appendiculata*.

Against this research background, the present study employed pseudobulbs of *C. appendiculata* as the primary experimental material and applied inoculants of three companion fungi, namely *C. micaceus*, *C. radians*, and *E. pseudodisseminata*, as treatments. Plant growth parameters, biomass accumulation, physiological and biochemical characteristics, and the contents of medicinal quality components including total flavonoids, total phenolics, polysaccharides, and alkaloids were systematically measured. The comprehensive effects of different companion fungi were evaluated using principal component analysis (PCA) and correlation analysis. The objectives of this study were to elucidate the differential regulatory effects of various companion fungi on the growth, development, and quality formation of *C. appendiculata* pseudobulbs; to screen for superior fungal inoculants capable of synchronously promoting both pseudobulb growth and bioactive component accumulation; and to establish a multi-indicator comprehensive evaluation system for screening companion fungi, thereby providing a theoretical basis and technical support for the efficient cultivation and sustainable utilization of medicinal resources of *C. appendiculata*.

## 2. Results

### 2.1. Effects of Different Companion Fungi on the Growth and Development of C. appendiculata Seedlings and Substance Accumulation in Pseudobulbs

**Figure 1:**
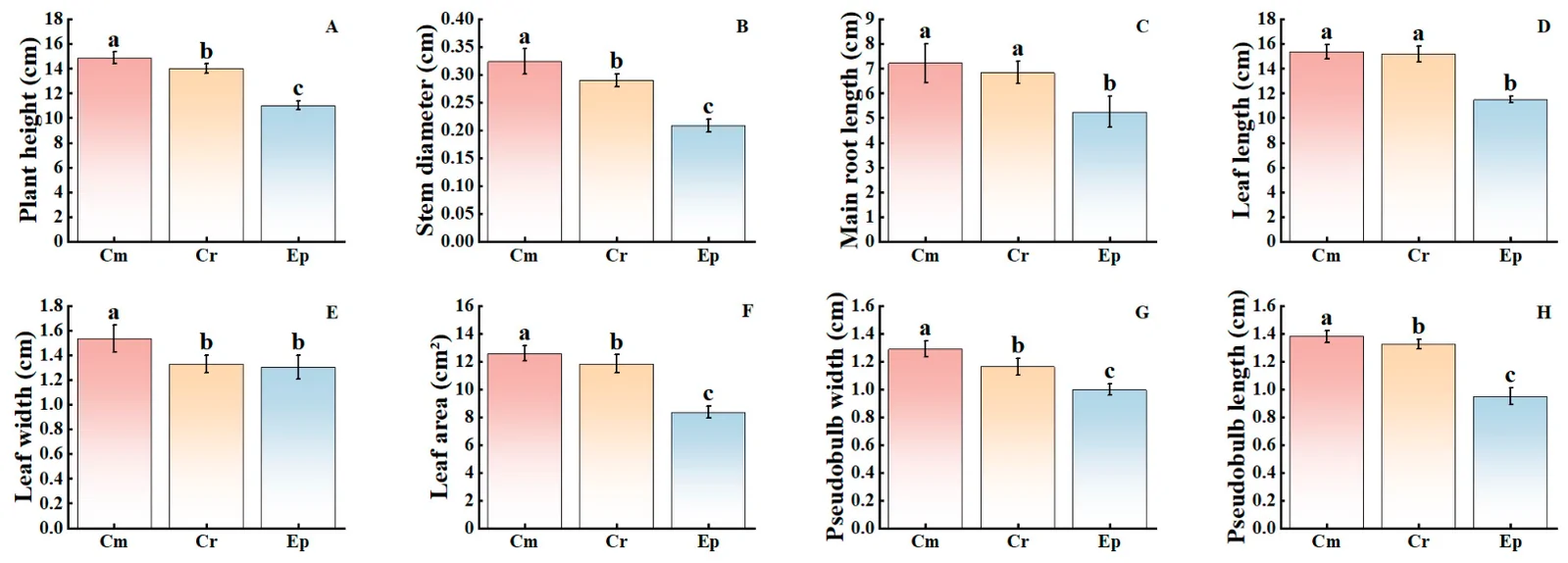
Effects of different companion fungi on the growth and development of *C. appendiculata* seedlings. (**A**) Plant height; (**B**) Stem diameter; (**C**) Main root length; (**D**) Leaf length; (**E**) Leaf width; (**F**) Leaf area; (**G**) Pseudobulb length; (**H**) Pseudobulb width. Cm: *Coprinellus micaceus*; Cr: *Coprinellus radians*; Ep: *Ephemerocybe pseudodisseminata*. Values are means ± SD (*n* = 9). Different letters indicate significant differences (*p* < 0.05).

**Figure 2:**
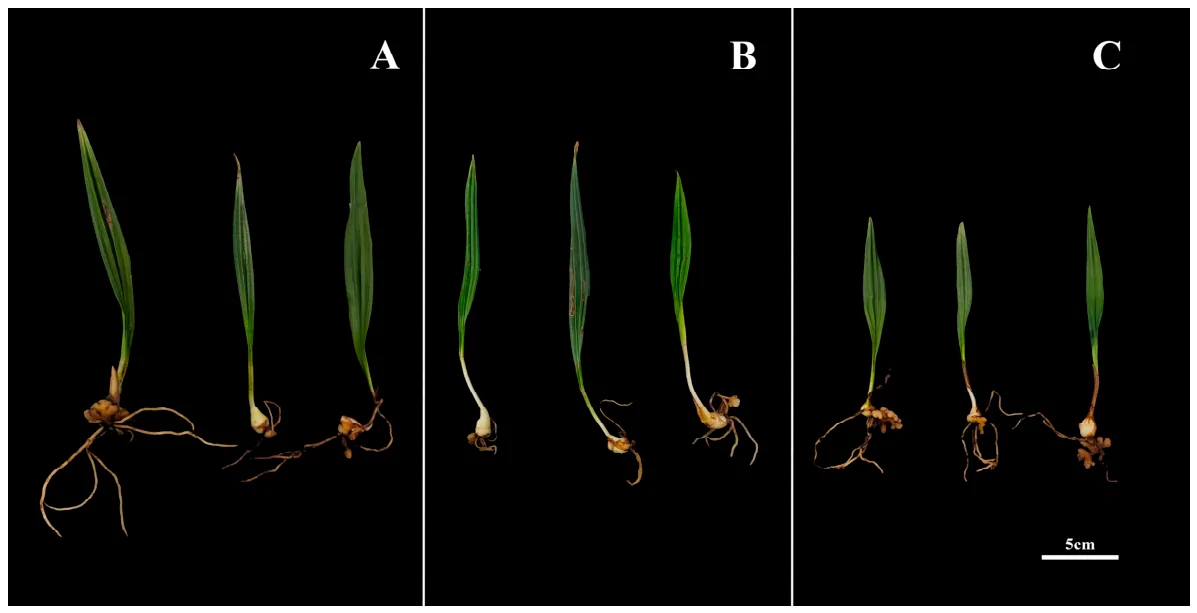
Seedlings of Cremastra appendiculata under different companion fungal inoculant treatments. (**A**) *Coprinellus micaceus*; (**B**) *Coprinellus radians*; (**C**) *Ephemerocybe pseudodisseminata*. These representative images illustrate the morphological differences among the various processing methods, while the quantitative growth parameters are shown in Figure 1.

Treatments with different companion fungal inoculants all exerted significant effects on the growth and development of *C. appendiculata* seedlings and substance accumulation in pseudobulbs (Figure 1). Regarding growth parameters, the *C. micaceus* treatment showed the highest values among all treatments. Its plant height (14.88 cm) and stem base diameter (0.32 cm) were significantly higher than those of the other treatments (*p* < 0.05), representing increases of 35.03% and 52.38%, respectively, compared to the *E. pseudodisseminata* treatment, which performed the poorest. In terms of leaf growth, the *C. micaceus* treatment also yielded the highest values among all treatments for leaf length (15.35 cm), leaf width (1.54 cm), and leaf area (12.58 cm^2^), with increases of 33.36%, 18.46%, and 50.47%, respectively, over the *E. pseudodisseminata* treatment. Regarding root growth, the main root length in the *C. micaceus* treatment (7.22 cm) did not differ significantly from that in the *C. radians* treatment (6.84 cm), but both were significantly greater than that in the *E. pseudodisseminata* treatment. In terms of pseudobulb morphology, both the length (1.38 cm) and width (1.29 cm) of pseudobulbs in the *C. micaceus* treatment were significantly higher than those in the other treatments, representing increases of 45.26% and 29.00%, respectively, compared to the *E. pseudodisseminata* treatment. The representative morphological differences among seedlings under the three treatments are shown in Figure 2. Overall, the relative performance of the different companion fungal inoculants on seedling growth of *C. appendiculata* ranked as follows: *C. micaceus* > *C. radians* > *E. pseudodisseminata*.

**Figure 3:**
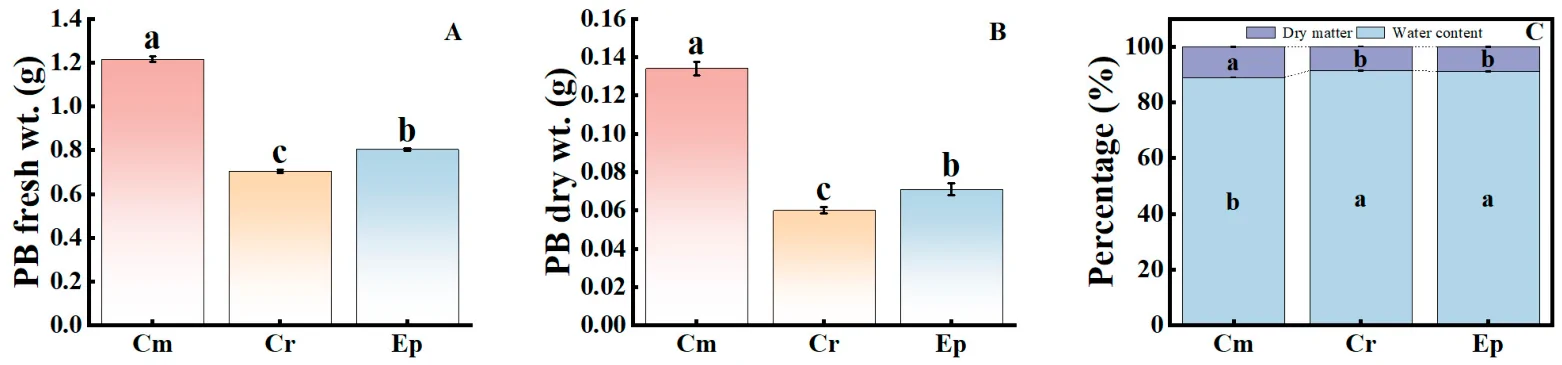
Effects of different companion fungi on substance accumulation in pseudobulbs of *C. appendiculata*. (**A**) Pseudobulb fresh weight; (**B**) Pseudobulb dry weight; (**C**) Proportions of water and dry matter in pseudobulbs. Cm: *Coprinellus micaceus*; Cr: *Coprinellus radians*; Ep: *Ephemerocybe pseudodisseminata*. Values are means ± SD (*n* = 9). Different letters indicate significant differences (*p* < 0.05).

In terms of substance accumulation in pseudobulbs (Figure 3), the fresh weight (1.22 g) and dry weight (0.13 g) of pseudobulbs in the *C. micaceus* treatment were significantly higher than those in the other treatments (*p* < 0.05), representing increases of 74.29% and 116.67%, respectively, compared to the *C. radians* treatment. Regarding water content, the *C. micaceus* treatment (88.97%) was significantly lower than the *C. radians* (91.40%) and *E. pseudodisseminata* (91.13%) treatments, while the dry matter content was highest in the *C. micaceus* treatment (11.03%), with no significant difference observed between the *C. radians* (8.60%) and *E. pseudodisseminata* (8.87%) treatments.

Collectively, these results indicate that different companion fungi exert significantly differential regulatory effects on the growth, development, and substance accumulation in pseudobulbs of *C. appendiculata*: *C. micaceus* showed the highest values for plant growth and biomass accumulation among the three treatments, while also demonstrating a certain advantage in dry matter accumulation.

### 2.2. Effects of Different Companion Fungi on the Physiological and Biochemical Characteristics of C. appendiculata Pseudobulbs

**Figure 4:**
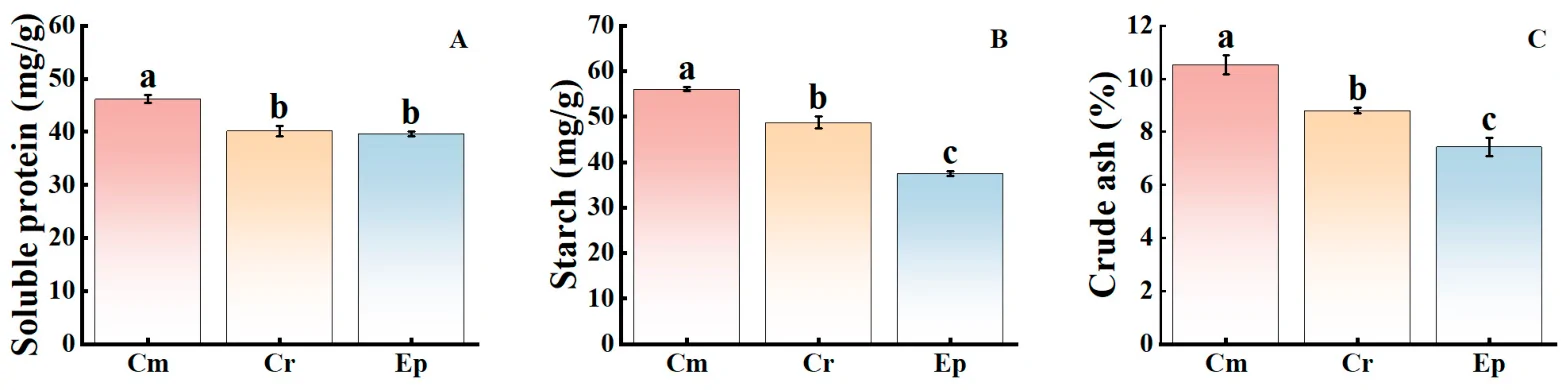
Effects of different companion fungi on the physiological and biochemical characteristics of *C. appendiculata* pseudobulbs. (**A**) Soluble protein content; (**B**) Starch content; (**C**) Crude ash content. Cm: *Coprinellus micaceus*; Cr: *Coprinellus radians*; Ep: *Ephemerocybe pseudodisseminata*. Values are means ± SD (*n* = 9). Different letters indicate significant differences (*p* < 0.05).

Treatments with different companion fungal inoculants significantly affected the contents of soluble protein, starch, and crude ash in pseudobulbs of *C. appendiculata* (Figure 4). The soluble protein content ranged from 39.62 to 46.17 mg·g^−1^, with the highest value observed in the *C. micaceus* treatment (46.17 mg·g^−1^), which was significantly higher than those in the *C. radians* and *E. pseudodisseminata* treatments, representing increases of 14.93% and 16.53%, respectively. The starch content was also highest in the *C. micaceus* treatment (56.08 mg·g^−1^), significantly exceeding those in the *C. radians* and *E. pseudodisseminata* treatments, with an increase of 49.51% compared to the *E. pseudodisseminata* treatment; a significant difference was also observed between the *C. radians* (48.72 mg·g^−1^) and *E. pseudodisseminata* (37.51 mg·g^−1^) treatments. The crude ash content was highest in the *C. micaceus* treatment (10.53%), significantly higher than in the other treatments, representing a 57.14% increase over the *E. pseudodisseminata* treatment; the crude ash content in the *C. radians* treatment (8.80%) was significantly higher than that in the *E. pseudodisseminata* treatment (7.43%).

Overall, nutrient accumulation was highest under *C. micaceus* treatment among the three treatments in pseudobulbs, particularly excelling in the accumulation of soluble protein, starch, and mineral elements.

### 2.3. Effects of Different Companion Fungi on the Accumulation of Bioactive Components in C. appendiculata Pseudobulbs

**Figure 5:**
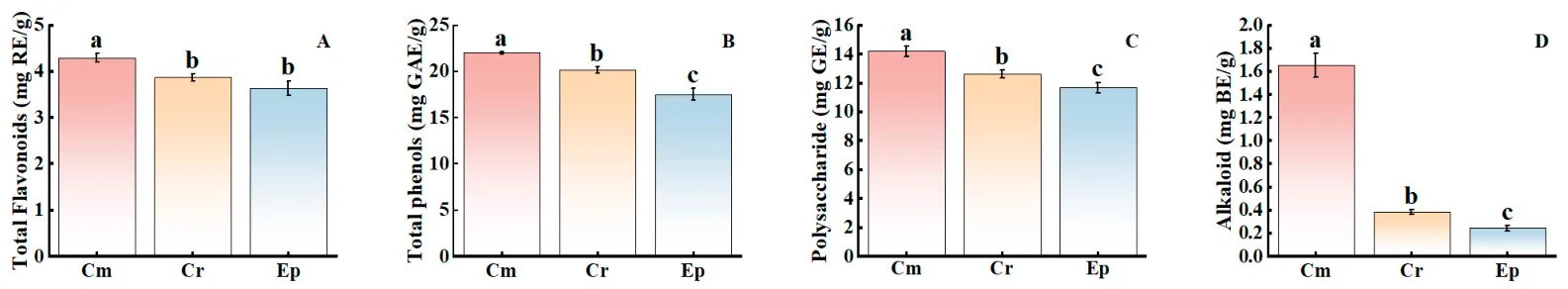
Effects of Different Companion Fungi on the Accumulation of Bioactive Components in *C. appendiculata* Pseudobulbs. (**A**) Total flavonoid content; (**B**) Total phenolic content; (**C**) Polysaccharide content; (**D**) Alkaloid content. Cm: *Coprinellus micaceus*; Cr: *Coprinellus radians*; Ep: *Ephemerocybe pseudodisseminata*. Values are means ± SD (*n* = 9). Different letters indicate significant differences (*p* < 0.05).

Treatments with different companion fungal inoculants significantly affected the contents of total flavonoids, total phenolics, polysaccharides, and alkaloids in pseudobulbs of *C. appendiculata* (Figure 5). The total flavonoid content ranged from 3.64 to 4.30 mg RE·g^−1^, with the highest value observed in the *C. micaceus* treatment (4.30 mg RE·g^−1^), which was significantly higher than those in the *C. radians* and *E. pseudodisseminata* treatments (*p* < 0.05), representing an 18.13% increase over the *E. pseudodisseminata* treatment; no significant difference was detected between the *C. radians* (3.86 mg RE·g^−1^) and *E. pseudodisseminata* (3.64 mg RE·g^−1^) treatments. The variation trend of total phenolic content was similar to that of total flavonoids, with the *C. micaceus* treatment (22.03 mg GAE·g^−1^) showing significantly higher values than the other two treatments, representing a 25.60% increase over the *E. pseudodisseminata* treatment; the *C. radians* treatment (18.78 mg GAE·g^−1^) was also significantly higher than the *E. pseudodisseminata* treatment (17.54 mg GAE·g^−1^). Regarding polysaccharide content, the *C. micaceus* treatment (14.20 mg GE·g^−1^) again exhibited the highest value, representing a 21.47% increase over the *E. pseudodisseminata* treatment; the *C. radians* treatment (12.65 mg GE·g^−1^) ranked intermediate and differed significantly from the *E. pseudodisseminata* treatment (11.69 mg GE·g^−1^). Notably, the alkaloid content was most dramatically affected by companion fungal treatments among all measured indices: the alkaloid content in the *C. micaceus* treatment reached as high as 1.66 mg BE·g^−1^, representing a 6.64-fold increase over the *E. pseudodisseminata* treatment and a 5.19-fold increase over the *C. radians* treatment; meanwhile, no significant difference was observed between the *C. radians* (0.32 mg BE·g^−1^) and *E. pseudodisseminata* (0.25 mg BE·g^−1^) treatments.

Taken together, the contents of medicinal bioactive components were highest under *C. micaceus* treatment in pseudobulbs of *C. appendiculata*, with a particularly pronounced inducing effect on alkaloid accumulation.

### 2.4. Principal Component Analysis and Correlation Analysis

To comprehensively evaluate the effects of different companion fungi on the growth and development, physiological and biochemical characteristics, and bioactive component accumulation in pseudobulbs of *C. appendiculata*, principal component analysis (PCA) and Pearson correlation analysis were performed on 19 indicators.

#### 2.4.1. Principal Component Analysis

**Figure 6:**
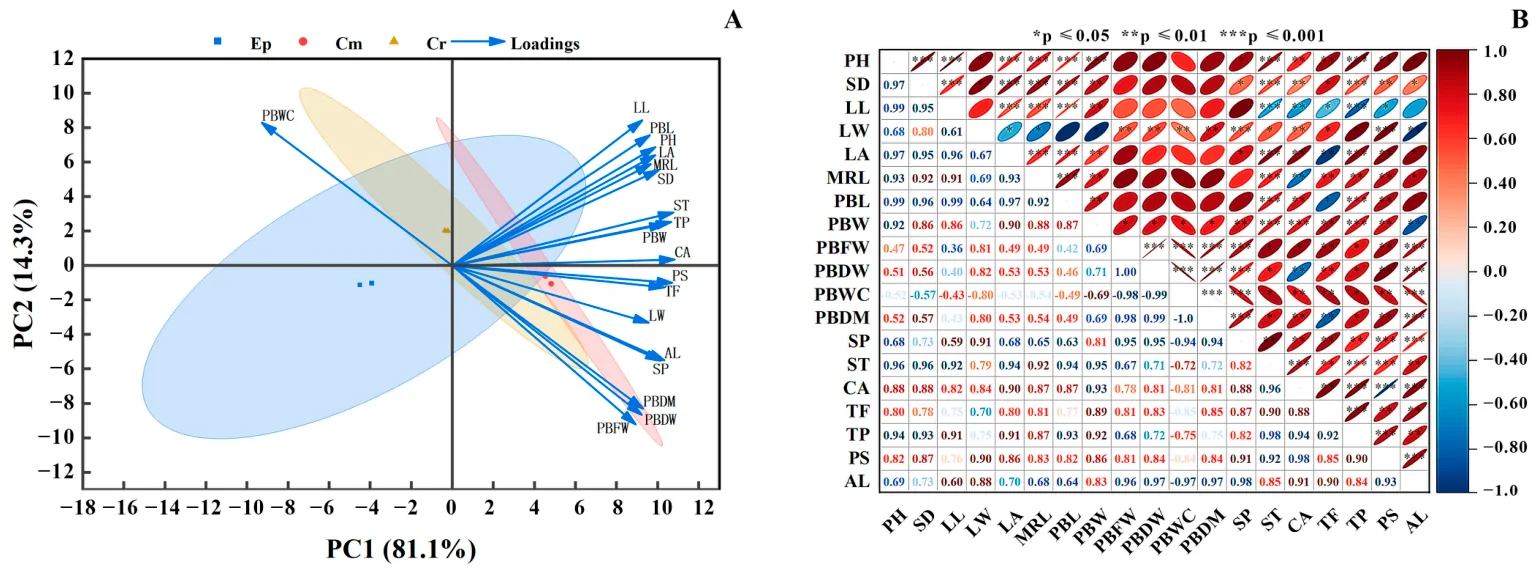
Principal component analysis (**A**) and correlation heatmap (**B**) of growth indicators of *C. appendiculata* seedlings, physiological and biochemical parameters of pseudobulbs, and bioactive components under different companion fungal treatments. PH: Plant height; SD: Stem base diameter; LL: Leaf length; LW: Leaf width; LA: Leaf area; MRL: Main root length; PBL: Pseudobulb length; PBW: Pseudobulb width; PBFW: Pseudobulb fresh weight; PBDW: Pseudobulb dry weight; PBWC: Pseudobulb water content; PBDM: Pseudobulb dry matter; SP: Soluble protein; ST: Starch; CA: Crude ash; TF: Total flavonoids; TP: Total phenolics; PS: Polysaccharides; AL: Alkaloids. In (**A**), the shaded ellipses represent the 95% confidence intervals for each treatment group.

The PCA results based on the correlation matrix (Figure 6A) showed that PC1 (81.1%) and PC2 (14.3%) cumulatively explained 95.4% of the total variance, effectively reflecting the comprehensive differences among treatments. Among the indicators, 15 parameters—including plant height, stem base diameter, leaf length, leaf width, leaf area, main root length, pseudobulb length and width, fresh weight, dry weight, soluble protein, starch, total flavonoids, total phenolics, polysaccharides, and alkaloids—were all concentrated in the positive direction of PC1, indicating that PC1 primarily reflected the growth and development level as well as the medicinal quality accumulation capacity of *C. appendiculata* pseudobulbs, serving as the core dimension for distinguishing the growth-promoting effects of different companion fungal inoculants. Pseudobulb water content was mainly distributed in the positive direction of PC2, while dry matter content was located in the negative direction of PC2, suggesting that PC2 primarily reflected differences in internal substance allocation and water status within pseudobulbs.

In terms of sample distribution, the different companion fungal treatments exhibited clear separation in the two-dimensional space formed by PC1 and PC2. The *C. micaceus* treatment was concentrated in the positive region of PC1, closely associated with most growth indicators and bioactive component parameters, demonstrating the best comprehensive performance. The *C. radians* treatment was positioned near the origin of the coordinate axes, with all indicators showing intermediate performance. The *E. pseudodisseminata* treatment was mainly distributed in the negative region of PC1, exhibiting negative correlations with most indicators and displaying the weakest comprehensive performance.

Based on the comprehensive score results, the overall promoting effects of different companion fungi on pseudobulbs of *C. appendiculata* ranked as follows: *C. micaceus* > *C. radians* > *E. pseudodisseminata*. This result indicates that *C. micaceus* possesses the optimal comprehensive effect in simultaneously promoting the growth and development, substance accumulation, and bioactive component formation of *C. appendiculata* pseudobulbs.

#### 2.4.2. Correlation Analysis

The correlation analysis results (Figure 6B) revealed that most growth indicators were significantly or extremely significantly positively correlated with quality indicators. Specifically, plant height, stem base diameter, leaf area, main root length, and pseudobulb length and width were all significantly positively correlated with pseudobulb fresh weight and dry weight (*p* < 0.05), indicating a high degree of synergy between plant morphological development and pseudobulb biomass accumulation, which is consistent with the composition of PC1 in the PCA. Pseudobulb fresh weight and dry weight were significantly positively correlated with soluble protein, starch, polysaccharide, and alkaloid contents, suggesting that the accumulation of nutrients provides a material foundation for the synthesis of secondary metabolites. Total flavonoids, total phenolics, polysaccharides, and alkaloids generally exhibited positive correlations with one another, among which total phenolics showed relatively high correlations with total flavonoids and polysaccharides, indicating that these secondary metabolites may be regulated by similar metabolic pathways. Pseudobulb water content exhibited a clear negative correlation with dry matter content, while dry matter content was positively correlated with total phenolics, polysaccharides, and alkaloid contents, suggesting that higher dry matter accumulation is conducive to the enrichment of medicinal bioactive components.

Integrating the results of principal component analysis and correlation analysis, pseudobulb fresh weight, dry weight, starch, polysaccharide, and alkaloid contents can serve as core indicators for evaluating the quality of *C. appendiculata* pseudobulbs. There was no apparent antagonism between growth promotion and quality formation; rather, companion fungus-mediated enhancement of nutrient accumulation may provide a more abundant carbon skeleton and energy source for secondary metabolism, thereby synergistically promoting the synthesis of bioactive components such as flavonoids, polysaccharides, and alkaloids.

## 3. Discussion

### 3.1. Effects of Different Companion Fungi on Pseudobulb Growth and Substance Accumulation in C. appendiculata

The pseudobulb is an important nutrient storage organ and the main medicinal part of *C. appendiculata*, and its growth status directly affects the subsequent plant development and medicinal material yield. In this study, the *C. micaceus* treatment performed best in plant height, stem base diameter, leaf area, main root length, and pseudobulb length and width, and also significantly increased pseudobulb fresh and dry weights, indicating that plants treated with this companion fungus showed the highest vegetative growth and assimilate translocation among the three treatments to the storage organ. Li et al. [14] found in Bletilla striata that the isolated *Tulasnella* sp. BJ1 strain increased the fresh weight of symbiotic protocorms to 3.7 times that of the asymbiotic group, which is consistent with our results and confirms that specific mycorrhizal fungi can significantly promote biomass accumulation in orchids. Shi et al. [10] further showed that hyphae of *C. micaceus* can be continuously digested by *C. appendiculata* protocorms, releasing nutrients and providing additional carbon and energy sources to the host, which partly explains why *C. micaceus* showed better performance than the other companion fungi.

Notably, the *C. micaceus* treatment produced the highest biomass and also had the highest dry matter content (11.03%), which was significantly higher than that of the *C. radians* (8.60%) and *E. pseudodisseminata* (8.87%) treatments. This suggests that *C. micaceus* not only promoted overall biomass production but also enhanced dry matter accumulation in pseudobulbs, indicating a more efficient assimilate partitioning toward the storage organ. In contrast, *C. radians* and *E. pseudodisseminata* showed lower dry matter accumulation, suggesting that these two fungi may direct more assimilates to aboveground growth rather than to pseudobulb storage. Zhang et al. [15] found in Pleione aurita pseudobulbs that the composition of non-structural carbohydrates (starch versus soluble sugars) changed markedly across developmental stages, indicating that carbon allocation in orchid pseudobulbs is highly plastic, which provides a reference for understanding the differential regulation of assimilate partitioning by different companion fungi.

### 3.2. Effects of Different Companion Fungi on Physiological and Biochemical Characteristics of C. appendiculata Pseudobulbs

Soluble protein and starch reflect the levels of nitrogen metabolism and carbon metabolism, respectively, and are important indicators for evaluating the nutrient accumulation capacity of medicinal plants [16]. In the present study, soluble protein and starch contents were significantly higher under the *C. micaceus* treatment, suggesting that levels of carbon and nitrogen metabolism were greater in *C. appendiculata* under this treatment. The concomitant increase in crude ash content further suggested that mineral element absorption and accumulation were higher under *C. micaceus* treatment. Nurfadilah et al. [17] demonstrated that orchid mycorrhizal fungi possess a broad capacity for utilizing various nutrient substrates, including inorganic and organic nitrogen sources as well as organic phosphorus sources (e.g., phytate), which may directly determine their efficiency in nutrient supply to the host. Transcriptomic analysis by Gao et al. [9] further confirmed at the molecular level that genes related to carbon metabolism, amino acid metabolism, and cell wall remodeling were significantly differentially expressed during the symbiotic interaction between *C. disseminatus* and *C. appendiculata*. The simultaneous significant increases in soluble protein and starch contents in pseudobulbs following *C. micaceus* treatment observed in this study are consistent with the conclusion that carbon and nitrogen metabolic pathways are broadly activated in the aforementioned transcriptomic findings.

Notably, the *C. micaceus* treatment significantly increased both fresh weight and dry weight of pseudobulbs, and its dry matter content (11.03%) was also significantly higher than that of the *C. radians* (8.60%) and *E. pseudodisseminata* (8.87%) treatments. This result indicates that *C. micaceus* enhanced both water uptake (contributing to fresh weight) and dry matter accumulation in pseudobulbs, resulting in a higher proportion of dry matter rather than a dilution effect. Although the present study did not conduct quantitative analysis of mycorrhizal colonization rate, the previous literature has reported that different fungi may exhibit considerable variation in colonization efficiency on the same host [1], and the number and longevity of hyphal pelotons formed within cortical cells directly affect nutrient exchange efficiency [18]. The dual advantages of *C. micaceus* in promoting both biomass accumulation and dry matter proportion suggest that it may possess a more efficient nutrient exchange interface; however, this hypothesis awaits verification through microscopic quantitative analysis of mycorrhizal colonization rates.

### 3.3. Regulatory Effects of Different Companion Fungi on Quality Component Accumulation in C. appendiculata Pseudobulbs

The pharmacological activities of *C. appendiculata* are primarily derived from secondary metabolites accumulated in pseudobulbs, including flavonoids, phenolics, polysaccharides, and alkaloids. In recent years, various compounds such as bibenzyls, phenanthrenes, and alkaloids have been identified from the pseudobulbs of *C. appendiculata*, and these constituents are closely associated with its anti-inflammatory, antioxidant, and antitumor activities [19]. In the present study, the contents of total flavonoids, total phenolics, polysaccharides, and alkaloids were significantly higher under *C. micaceus* treatment than under the other two treatments, among which the alkaloid content reached 1.66 mg BE·g^−1^, representing a 6.64-fold increase over the *E. pseudodisseminata* treatment and a 5.19-fold increase over the *C. radians* treatment. This enhancement is particularly notable among studies on companion fungi in medicinal plants. Billowria et al. [20] reported that treatment of hairy roots of *Gloriosa superba* with the fungal elicitor *Alternaria alternata* increased colchicine content by approximately 3.4-fold. The 6.64-fold increase in alkaloid content observed in this study substantially exceeds that level, suggesting that *C. micaceus* may be associated with a stronger eliciting effect on the alkaloid biosynthetic pathway compared with the other two fungi, possibly through distinct metabolic regulatory mechanisms.

Notably, total flavonoids, total phenolics, polysaccharides, and alkaloids exhibited a synergistic increasing trend in this study, indicating that the variation in these secondary metabolites among the three fungal treatments was not restricted to a single metabolic pathway, but rather involved the overall secondary metabolic network. This result is consistent with the findings of Lai et al. [13], who reported that microbial fertilizers can enhance the quality of medicinal plants by activating multiple secondary metabolic pathways, including the phenylpropanoid pathway.

Meanwhile, the alkaloid content under the *C. micaceus* treatment was 6.64-fold higher than that under the *E. pseudodisseminata* treatment, an increment far exceeding the relative differences observed for total flavonoids (18.13%) and total phenolics (25.60%), indicating that *C. micaceus* may be specifically associated with the activation of the alkaloid biosynthetic pathway in *C. appendiculata*, rather than a non-selective effect on the entire secondary metabolic network. Fungal elicitors activate plant secondary metabolism with a high degree of specificity; different microorganisms and their associated signaling molecules (such as chitin oligosaccharides, lipopolysaccharides, and volatile organic compounds) may selectively activate distinct defense signaling pathways in plants, thereby differentially regulating the expression of various secondary metabolic branches [21]. As nitrogen-containing secondary metabolites, alkaloids depend not only on the activities of key enzymes in the shikimic acid and mevalonate pathways (e.g., tryptophan decarboxylase, N-methyltransferases) but are also closely related to nitrogen assimilation and the coordination of carbon–nitrogen metabolism in plants. In this study, the soluble protein content was simultaneously higher under *C. micaceus* treatment (by 16.53% compared with the *E. pseudodisseminata* treatment), suggesting that the higher nitrogen metabolism level in this treatment may provide a more abundant nitrogen source and amino precursors for alkaloid biosynthesis. Furthermore, Li et al. [14] demonstrated that different mycorrhizal fungi exhibit significant differences in the number and longevity of pelotons formed within host cortical cells, which may affect the efficiency and duration of signal molecule exchange between the fungus and the host, ultimately leading to differential activation of secondary metabolic pathways. Zhou et al. [22] reported in *Catharanthus roseus* that different fungal elicitors induced differential accumulation of indole alkaloids by more than 5-fold, and this variation was closely related to the intensity of jasmonic acid signaling induced by the elicitors. Similar phenomena have also been reported in medicinal orchids: Chen et al. [23] found that fungal elicitors differentially activated key enzymes (PAL, C4H, 4CL) in the phenylpropanoid pathway of Anoectochilus roxburghii, leading to significant differences in flavonoid accumulation; Zhang et al. [24] reported that the effects of different Armillaria strains on gastrodin content in Gastrodia elata were related to fungal-produced IAA and ABA levels, suggesting that fungal hormone signals play an important role in regulating secondary metabolism. Based on the above literature and the indirect evidence from this study, we speculate that *C. micaceus* may activate defense signaling pathways such as jasmonic acid or salicylic acid in *C. appendiculata* through specific signaling molecules, thereby specifically inducing the upregulation of key genes in the alkaloid biosynthetic pathway and ultimately leading to the higher alkaloid content observed in this treatment. It should be noted that the above mechanistic speculation is mainly based on indirect evidence at the physiological level and has not yet been directly verified at the molecular regulatory level. Future studies may further elucidate the molecular mechanisms by which companion fungi regulate alkaloid accumulation in *C. appendiculata* through transcriptomic, metabolomic, and signaling pathway analyses.

### 3.4. Screening of Superior Companion Fungi Based on Multi-Indicator Comprehensive Evaluation

In studies on the screening of companion fungi for medicinal plants, traditional evaluation methods typically rely on plant height, biomass, or a single bioactive component as the assessment criterion. However, for *C. appendiculata*, whose pseudobulbs serve as the medicinal organ, a single indicator is often insufficient to comprehensively reflect the process of medicinal material quality formation. Previous studies have demonstrated that the quality formation of medicinal plants results from the combined effects of primary metabolism and secondary metabolism [25]. Pseudobulb fresh weight, dry weight, and starch accumulation reflect the plant’s capacity for acquisition and storage of photosynthetic products, whereas bioactive components such as flavonoids, phenolics, polysaccharides, and alkaloids represent the intrinsic quality level of the medicinal material [26]. Although these two categories of indicators are closely related, they do not always change synchronously. Therefore, screening companion fungi based solely on a single growth indicator or quality indicator cannot accurately reflect their comprehensive effects on both plant growth and medicinal quality.

In the principal component analysis of this study, the cumulative contribution rate of PC1 and PC2 reached 95.4%, indicating that the established evaluation system effectively captured most of the variation information among different companion fungal treatments. PC1 was predominantly composed of pseudobulb growth, biomass accumulation, and bioactive component contents, suggesting that the key factor associated with the quality differences in *C. appendiculata* is the synergistic variation in both growth and development capacity and metabolic accumulation capacity, rather than a single indicator. The ranking of treatments on PC1 was *C. micaceus* > *C. radians* > *E. pseudodisseminata*, which is highly consistent with the performance of each treatment in key indicators such as soluble protein, starch, polysaccharides, and alkaloids, thereby validating the effectiveness of the comprehensive evaluation system. Furthermore, correlation analysis revealed that most growth indicators were generally positively correlated with bioactive component indicators, indicating that during the pseudobulb development stage of *C. appendiculata*, there is no apparent antagonistic relationship between growth promotion and quality formation. Following companion fungus-mediated enhancement of nutrient accumulation, a greater supply of carbon skeletons and energy sources may be provided for secondary metabolism, thereby further promoting the synthesis of bioactive components.

## 4. Materials and Methods

### 4.1. Description of the Experimental Site

The experimental site was located at the Entrepreneurship Incubation Demonstration Base of Hongshi Forestry Bureau, Hongshilazi Town, Huadian City, Jilin Province, China (126°42′ E, 42°57′ N). The region has a northern temperate continental monsoon climate with four distinct seasons, characterized by an annual mean temperature of 3.9 °C, an annual mean sunshine duration of 2379 h, and an annual mean precipitation of 748.1 mm. The soil at the experimental site was classified as dark brown forest soil, with a pH of 5.6, organic matter content of 34.9 g·kg^−1^, total nitrogen of 0.236 g·kg^−1^, total phosphorus of 0.075 g·kg^−1^, total potassium of 1.83 g·kg^−1^, available nitrogen of 190 mg·kg^−1^, available phosphorus of 5 mg·kg^−1^, and available potassium of 158 mg·kg^−1^.

### 4.2. Experimental Design

Since *C. appendiculata* seeds cannot germinate under sterile conditions [4], a sterile blank control group was not established in this study. To verify the applicability of this conclusion under our experimental conditions, we conducted a pre-experiment in which *C. appendiculata* seeds were mixed with sterile substrate (without any fungal inoculant) and incubated under the same conditions. The results showed a germination rate of 0%, further confirming that *C. appendiculata* seeds cannot complete germination and seedling establishment under sterile conditions. Therefore, this study focused on comparing the differential regulatory effects of different fungi on plant growth, physiological metabolism, and medicinal quality formation during the pseudobulb developmental stage, with treatments compared against each other as mutual controls, aiming to identify the superior fungal inoculant with the best comprehensive performance.

The experiment commenced in early December 2024. To shorten the seedling propagation cycle and achieve standardized control during the seed germination stage, an incubator was employed to induce symbiotic seed germination. First, plump and healthy capsules of *C. appendiculata* were selected and surface-sterilized, after which the capsules were dissected under sterile conditions to collect seeds. The seeds were evenly spread onto sterile paper, and a clean scraper was used to disperse the seeds uniformly to avoid clumping. Seed samples corresponding to a coverage area of 50 cm^2^ (approximately 15,000 seeds, estimated at 300 seeds·cm^−2^) were separately and thoroughly mixed with three different solid fungal inoculants, namely *C. micaceus*, *C. radians*, and *E. pseudodisseminata* (3000 g of inoculant per 50 cm^2^ of seeds). The mixtures were placed into sterile 20 × 30 cm inoculant bags and incubated in darkness at 25 °C to induce symbiotic seed germination [10].

The three solid fungal inoculants were all purchased from Jinlong Edible Fungus Research Institute, Huadian City, Jilin Province, China. In this study, *Coprinellus micaceus*, *Coprinellus radians*, and *Ephemerocybe pseudodisseminata* were selected as the test fungi based on the following criteria. First, all three fungi have been previously confirmed to promote seed germination and seedling establishment of *C. appendiculata* [1,4,10], providing a clear functional basis for their selection. Second, we isolated multiple fungal strains from the rhizosphere of *C. appendiculata* and selected three fungi with high isolation frequency and functional representativeness for subsequent comparative study. Molecular identification of the three fungi was performed by Sangon Biotech (Shanghai) Co., Ltd. Fungal genomic DNA was extracted using the Ezup Column Fungal Genomic DNA Extraction Kit (Sangon Biotech (Shanghai) Co., Ltd., Shanghai, China). PCR amplification was conducted with ITS1 (TCCGTAGGTGAACCTGCGG)/ITS4 (TCCTCCGCTTATTGATATGC) and NS1 (GTAGTCATATGCTTGTCTC)/NS6 (GCATCACAGACCTGTTATTGCCTC) primers. The PCR products were detected by agarose gel electrophoresis, purified by gel extraction, and sequenced using an ABI 3730XL sequencer (Applied Biosystems, Foster City, CA, USA). BLAST analysis using BLAST+ 2.17.0 against the NCBI database showed that the *C. micaceus* strain had 100% homology with *Coprinellus micaceus*, the *C. radians* strain had 99.80% homology with *Coprinellus radians*, and the *E. pseudodisseminata* strain had 100% homology with *Ephemerocybe pseudodisseminata*.

After seed germination and protocorm formation, i.e., in mid-April 2025, protocorms with transverse diameters of 2–3 mm were selected and uniformly sown at a density of approximately 800 plants·m^−2^ into artificially constructed rectangular seedbeds measuring 3 m in length and 1 m in width. The seedbed substrate consisted of a mixture of fungal inoculant and soil (1:1, *v*/*v*). Each companion fungal inoculant treatment was replicated in three seedbeds, resulting in a total of nine independent seedbed units. Immediately after sowing, a black shade net with 75% shading rate was applied for shading treatment, and the shade net was fixed on a support frame 2 m above the seedbeds to ensure uniform light conditions across all treatments. On a clear day during the early stage of the experiment, at 10:00 a.m., the photosynthetic photon flux density (PPFD) was measured using a LI-190R quantum sensor (LI-COR Biosciences, Lincoln, NE, USA), with an average value of 300–350 µmol·m^−2^·s^−1^. Throughout the growth period, irrigation management was kept consistent across treatments, and no fertilizer was applied.

Sampling was conducted in early September 2025 for each treatment. The entire experimental period, from the initiation of symbiotic germination to the completion of sampling, lasted a total of 9 months.

### 4.3. Sample Collection and Measurement Methods

#### 4.3.1. Determination of Seedling Growth and Development Parameters and Pseudobulb Substance Accumulation Indicators

A total of 27 seedling samples were selected in this experiment, with 3 *C. appendiculata* seedlings randomly chosen from each seedbed (i.e., 9 seedlings per treatment, derived from three independent seedbed replicates), and their growth and development parameters as well as pseudobulb substance accumulation indicators were measured.

Plant height, stem base diameter, main root length, and pseudobulb length and width were measured using a ruler and vernier calipers. Leaf length, leaf width, and leaf area were measured using an AM-300 handheld leaf area meter (Zhejiang Top Cloud-Agri Technology Co., Ltd., Hangzhou, China) to scan the leaves. After measurement, the pseudobulbs were excised and their fresh weight was recorded. Following weighing, the pseudobulbs were placed in paper boxes and oven-dried at 105 °C for 30 min to inactivate enzymes, after which the temperature was adjusted to 65 °C and drying continued until constant weight was achieved. The dry weight of pseudobulbs was measured using an analytical balance (Sartorius Scientific Instruments (Beijing) Co., Ltd., Beijing, China), and the water content and dry matter content of pseudobulbs were calculated [27]. The calculation formulas were as follows:

Water content (%) = (Fresh weight − Dry weight)/Fresh weight × 100%

Dry matter content (%) = Dry weight/Fresh weight × 100%

#### 4.3.2. Determination of Physiological and Biochemical Indicators of Pseudobulbs

A total of 90 seedling samples were selected in this experiment, with 10 *C. appendiculata* seedlings randomly chosen from each seedbed (i.e., 30 seedlings per treatment, derived from three independent seedbed replicates). The pseudobulbs were placed in paper boxes and oven-dried at 105 °C for 30 min to inactivate enzymes, after which the temperature was adjusted to 65 °C and drying continued until constant weight was achieved. Finally, the dried pseudobulb samples were separately ground, passed through a 60-mesh sieve, and the resulting dry powder samples were collected for the determination of physiological, biochemical, and bioactive component indicators.

Soluble protein content was determined using the Coomassie Brilliant Blue G-250 staining method [28], with bovine serum albumin (Shanghai Macklin Biochemical Co., Ltd., Shanghai, China) used as the standard to generate a standard curve, and absorbance was measured at 595 nm. Starch content was determined using the anthrone–sulfuric acid colorimetric method [29], with absorbance measured at 620 nm. Crude ash content was determined according to the method described in GB 5009.4—2016 [30], using a muffle furnace (Shanghai Muffle Furnace Scientific Instrument Co., Ltd., Shanghai, China) at 550 °C until constant weight was achieved, followed by weighing and calculation.

#### 4.3.3. Determination of Bioactive Component Indicators in Pseudobulbs

The dried powder samples of *C. appendiculata* pseudobulbs prepared in Section 4.3.2 were used for the determination of bioactive component indicators. Total flavonoid content was determined using the aluminum nitrate–sodium nitrite colorimetric method [21], with rutin (Shanghai Macklin Biochemical Co., Ltd., Shanghai, China) used as the standard to generate a standard curve, and absorbance was measured at 510 nm. The results were expressed as rutin equivalents (mg RE·g^−1^). Total phenolic content was determined using the Folin–Ciocalteu colorimetric method [21], with gallic acid (Shanghai Macklin Biochemical Co., Ltd., Shanghai, China) used as the standard to generate a standard curve, and absorbance was measured at 760 nm. The results were expressed as gallic acid equivalents (mg GAE·g^−1^). Polysaccharide content was determined using the phenol–sulfuric acid method [31], with glucose (Shanghai Macklin Biochemical Co., Ltd., Shanghai, China) used as the standard to generate a standard curve, and absorbance was measured at 490 nm. The results were expressed as glucose equivalents (mg GE·g^−1^). Alkaloid content was determined using the acid dye colorimetric method [32], with berberine hydrochloride (Shanghai Macklin Biochemical Co., Ltd., Shanghai, China) used as the standard to generate a standard curve, and absorbance was measured at 416 nm. The results were expressed as berberine equivalents (mg BE·g^−1^).

### 4.4. Statistical Analysis

Experimental data were organized and preliminarily processed using Microsoft Excel 2020 (Microsoft Corporation, Redmond, WA, USA), and subsequent statistical analyses were performed using IBM SPSS Statistics 22 (IBM Corporation, Armonk, NY, USA). One-way analysis of variance (ANOVA) was employed to evaluate statistical significance among different companion fungal inoculant treatments, and Duncan’s multiple range test was used for multiple comparisons. Statistical differences between means (*p* < 0.05) were denoted by different letter labels (a, b, c). Graphical visualizations were created using OriginPro 2021 (OriginLab Corporation, Northampton, MA, USA).

Principal component analysis (PCA) was computed based on the correlation matrix. All indicators were subjected to Z-score standardization prior to PCA to ensure that each variable was assigned equal weight in the analysis. Correlation analysis was performed using Pearson’s correlation coefficient method (normality tests confirmed that the data for each indicator approximately followed a normal distribution) on the 19 indicators. Significance tests were conducted using a two-tailed test, with *p* < 0.05 considered statistically significant.

## 5. Conclusions

This study investigated the effects of different companion fungal treatments on the growth and development, physiological and biochemical characteristics, and medicinal quality component accumulation of *C. appendiculata* pseudobulbs. The main conclusions are as follows:

- Among the three companion fungal treatments, plants treated with *C. micaceus* had significantly higher plant height, stem base diameter, leaf area, main root length, and pseudobulb length and width than those treated with the other two fungi, and also showed the highest pseudobulb fresh and dry weights.
- The contents of soluble protein, starch, and crude ash in pseudobulbs were highest under *C. micaceus* treatment, indicating a relatively higher level of nutrient accumulation in this treatment.
- *C. micaceus* showed the most pronounced effect on the accumulation of medicinal bioactive components, with the contents of total flavonoids, total phenolics, polysaccharides, and alkaloids all reaching relatively high levels, among which the alkaloid content was significantly higher than that under the other treatments.
- The comprehensive evaluation based on principal component analysis ranked the treatments as follows: *C. micaceus* > *C. radians* > *E. pseudodisseminata*.

Based on the integrated analysis of growth, physiological, and quality indicators, among the three companion fungi tested, *C. micaceus* showed the best comprehensive performance, demonstrating promising application potential in seedling propagation and efficient cultivation of medicinal resources of *C. appendiculata*. The comprehensive evaluation system established in this study, based on growth indicators, physiological and biochemical parameters, and quality component indices, may provide a reference for screening superior companion fungi for medicinal orchids. Future studies may further integrate metabolomics, transcriptomics, and microbial interaction analyses to elucidate the molecular mechanisms by which companion fungi influence bioactive component accumulation in *C. appendiculata*.

## Data Availability

The original contributions presented in this study are included in the article. Further inquiries can be directed to the corresponding author.

## Abbreviations

The following abbreviations are used in this manuscript:

PCA	Principal component analysis
ANOVA	Analysis of variance
PPFD	Photosynthetic photon flux density
PH	Plant height
SD	Stem base diameter
LL	Leaf length
LW	Leaf width
LA	Leaf area
MRL	Main root length
PBL	Pseudobulb length
PBW	Pseudobulb width
PBFW	Pseudobulb fresh weight
PBDW	Pseudobulb dry weight
PBWC	Pseudobulb water content
PBDM	Pseudobulb dry matter
SP	Soluble protein
ST	Starch
CA	Crude ash
TF	Total flavonoids
TP	Total phenolics
PS	Polysaccharides
AL	Alkaloids
